# Food globalization in southern Central Asia: archaeobotany at Bukhara between antiquity and the Middle Ages

**DOI:** 10.1007/s12520-023-01827-z

**Published:** 2023-07-21

**Authors:** Basira Mir-Makhamad, Sören Stark, Sirojidin Mirzaakhmedov, Husniddin Rahmonov, Robert N. Spengler

**Affiliations:** 1 Department of Archaeology, Max Planck Institute of Geoanthropology, Jena, Germany; 2 Domestication and Anthropogenic Evolution Research Group, Max Planck Institute of Geoanthropology, Jena, Germany; 3 Ancient Oriental Studies Department, Friedrich Schiller University, Jena, Germany; 4 Institute for the Study of the Ancient World at New York University, New York, NY USA; 5 Samarkand Institute of Archaeology, Agency of Cultural Heritage of the Republic of Uzbekistan, Samarkand, Uzbekistan

**Keywords:** Archaeobotany, Paleoethnobotany, Transoxiana, Silk Road, Sumac, Qarakhanid, Bukhara, Afrasiab

## Abstract

The Silk Road is a modern name for a globalization phenomenon that marked an extensive network of communication and exchange in the ancient world; by the turn of the second millennium AD, commercial trade linked Asia and supported the development of a string of large urban centers across Central Asia. One of the main arteries of the medieval trade routes followed the middle and lower Zarafshan River and was connected by mercantile cities, such as Samarkand and Bukhara. Bukhara developed into a flourishing urban center between the fourth and sixth centuries AD, served as the capital of the Samanid court between AD 893 and 999, and remained prosperous into the Qarakhanid period (AD 999–1220), until the Mongol invasion in AD 1220. We present the first archaeobotanical study from this ancient center of education, craft production, artistic development, and commerce. Radiocarbon dates and an archaeological chronology that has been developed for the site show that our samples cover a range between the third and eleventh centuries AD. These samples from Bukhara represent the richest systematically collected archaeobotanical assemblage thus far recovered in Central Asia. The assemblage includes spices and both annual and perennial crops, which allowed Sogdians and Samanids to feed large cities in river oases surrounded by desert and arid steppe and supported a far-reaching commercial market in the first millennium AD.

**Supplementary Information:**

The online version contains supplementary material available at 10.1007/s12520-023-01827-z.

## Introduction

**Fig. 1:**
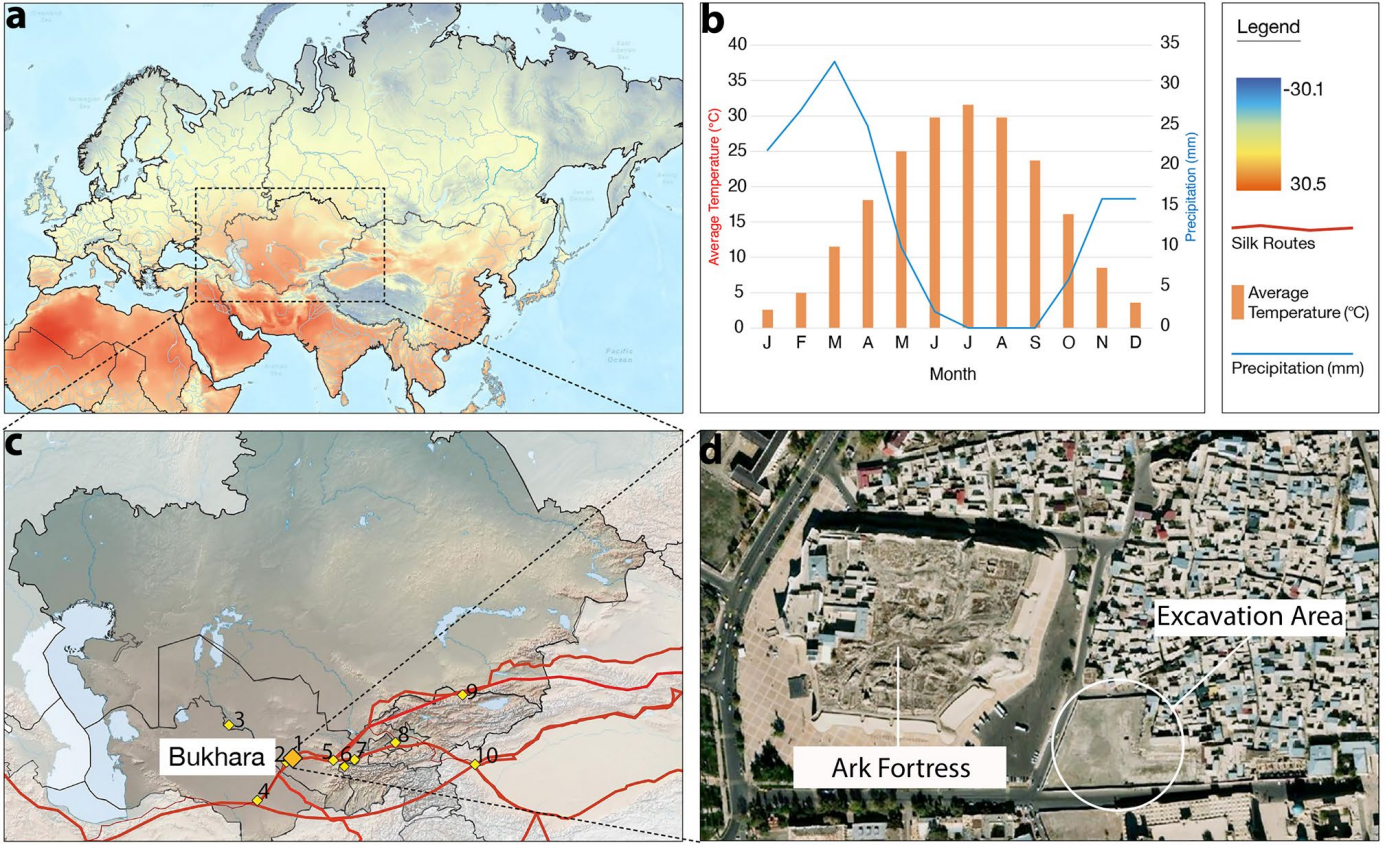
**a** Temperature gradients in Eurasia for Julys, averaged from 1950 to 2000 (figure was generated in ArcGIS using the dataset provided by Hijmans et al. 2005); **b** climate settings in Bukhara (data obtained from https://en.climate-data.org); **c** location of the core Silk Road cities in Central Asia: 1—Bukhara, 2—Paykend, 3—Khiva, 4—Merv, 5—Samarkand/Afrasiab, 6—Panjakent, 7—Tashbulak, 8—Kokand, 9—Suyab/Ak-Beshim, and 10—Kashgar; **d** aerial photo of the Bukhara medieval center (taken by Sirojidin Mirzaakhmedov)

For centuries, the region of Sogdiana in southern Central Asia, watered by the river systems of the Zarafshan and Kashka-Darya, held a key position in a complex network of commercial, political, and cultural exchange, that spanned across large parts of the ancient world. One of the linchpin cities of this network was Bukhara, known for having been a center of culture and trade in Central Asia since the first millennium AD, it is located in the wetland oasis formed by the delta of the Zarafshan River (Lo Muzio 2009; Rante and Mirzaakhmedov 2019). Archaeological research spanning close to a century demonstrates that a settlement existed on the spot by at least the early third century BC, but it seems to have turned into a proper city and consequently became the political center of the oasis only some point between the late third and sixth centuries AD (Stark and Mirzaakhmedov 2023). At the same time, investments in old and new feeder canals (Lurje 2006) led to an intensification of agricultural production and transformed the rural landscape with hundreds of new settlements, including numerous small, but economically vibrant rural towns across the delta of the Zarafshan River. The Islamic conquest of this rich region, starting in AD 706, was a protracted process, lasting several decades (Stark 2018), before the city eventually became a center of Islamic learning (Frye 1998). Archaeological and historical data demonstrate that there was a period of economic prosperity, when the city housed the court of the Samanid dynasty (AD 893–999), which since the end of the ninth and for most of the tenth centuries exerted suzerainty, not only over the rich provinces of Transoxiana (*mā-warā’-al-nahr*), but also over Tabaristan, Gurgan, Khorasan, Sistan, Guzgan, Tokharistan, and Khwarazm. Though the official status of the city changed with the establishment of Qarakhanid political authority (AD 999–1220), overthrowing the Samanid dynasty at the end of the tenth century AD, Bukhara nonetheless remained a key city of the Western Qarakhanid Qaghanate, and new palaces were constructed or added to previous ones (Karev 2013). Unlike Old-Samarkand (the present-day site of Afrasiab), Bukhara was not abandoned after the Mongol Conquest, but continued to be intensively inhabited on the same spot, which has rendered large-scale archaeological excavations in the present-day city center extremely rare (Fig. 1c).

Though the beginning of archaeological exploration of the oasis goes back to the end of the nineteenth century, when the region was still part of the Emirate of Bukhara, and though excavations in the inner city started as early as 1934, most of the earlier archaeological soundings where either very small or lacked present-day standards of documentation and detail, or both (Stark 2023; Shishkin 1955; Mukhamedjanov 1984). Only since 2020 — and for the first time in the history of archaeological research in the city — has a sizeable area (0.75 ha) within the inner city been investigated using modern standards of documentation and making full use of a wide range of archaeo-scientific methods of analysis, all within the framework of the Uzbek-American Expedition to Bukhara (UzAmEB). Notably, this includes archaeobotanical research — the results of which considerably advance our understanding not only of the broader culinary traditions found at ancient Silk Road cities (Spengler 2019a), but also specific socio-economic dynamics in the city and the wider region since the late third/fourth centuries AD. Some plant remains recovered in Bukhara are previously undocumented for the region.

Of course, it is already recognized that the period of political and economic prosperity towards the end of the first millennium AD in southern Central Asia was connected to agricultural development. However, the origins and sources of this prosperity are still subject to debate (Negmatov 1998). Watson (1974) claimed that agricultural diversification through introducing new crops and agricultural changes in Western Asia and North Africa prior to the seventh century AD was a result of farming transportations implemented by Islamic authorities. He states that new introductions were facilitated by political and demographic changes, serving as an auxiliary lever for the new farming and irrigation techniques. However, it is important to note that Watson does not exclude the fact that a number of crops discussed by him had been introduced prior to the Islamic conquest. Despite the fact that Watson’s hypothesis has subsequently been criticized by Decker (2009) and Squatriti (2014), there are a number of studies testing this concept through archaeobotany. For example, van der Veen and Morales (2017), comparing archaeobotanical data from Roman and Islamic deposits at the Quseir al-Qadim site, demonstrates that many crops were incorporated during the early Islamic period. Fuks and his colleagues (2020) introduced criteria for testing the Islamic Green Revolution thesis, calling for a greater focus on taphonomy, sampling and recovery, context and dating, taxonomic resolution of identification, geographic region, and historical evidence. However, more research at different archaeological sites is needed before we can clearly discuss the existence of an Islamic Green Revolution in Central Asia (Mir-Makhamad and Spengler 2023). Until recently, modern methods in the archaeological sciences have been lacking, leading to a poor understanding of what economy looked like. Using archaeobotanical remains from medieval waste and garbage pits and charred deposits in anthropogenic sediments, our major questions in this study are: (1) what were the economic plants used in ancient central Transoxiana in the first millennium AD; (2) when did agricultural systems diversify in the lower Zarafshan region in the first millennium AD; and (3) what role did the trade routes play in facilitating the introduction of new crops in the first millennium AD.

### Geographic and environmental setting

Bukhara (39° 46′ 36.228″ N, 64° 24′ 46.8396″ E; 225 masl) is located in the Bukhara Oasis in the Zarafshan River delta of central Uzbekistan. The oasis is bound by the Kyzyl-Kum Desert from the north, the Qara-Qum Desert from the south, and the Orta-Chul steppe to the east. The region today experiences a semiarid climate with hot summers (the monthly average in July is 31.6 °C) and cold winters (the monthly average in January is 2.6 °C). An annual average temperature of 17.1 °C and the annual precipitation is 157 mm (https://en.climate-data.org) (Fig. 1b). In Uzbekistan, around 37% of the precipitation falls as snow and 39% falls as rain in the spring (Gintzburger et al. 2003). The low precipitation rates, combined with extreme temperature fluctuations, lead to soil salinization (Shadyeva 2021). Despite the fact that today only 4.7% of the surface area of the Bukhara oasis is used for agriculture, farming is, for most regions, the basis of the economy, largely focused on the cash crops of silk and cotton, which feed a historically significant textile industry (Kulmatov et al. 2015).

The Zarafshan River is the main water source for agriculture in the region (Mukhamedzhanov 1978; Rante and Mirzaakhmedov 2019). Historically, the landscape of the Bukhara Oasis has been subject to cycles of expansion and contraction, due to changing anthropogenic and environmental factors occurring along the Zarafshan River (Lo Muzio 2009; Zink et al. 2017). The Bukhara Oasis experienced problems with irrigation during the Qarakhanid period (Tārīkh-i Bukhārā 1984), but localized aridification processes occurred much earlier, such as when the site-cluster around Bashtepa was abandoned around AD 100 (see also Stark and Mirzaakhmedov 2023; Stark et al. 2019).

## Methods and materials

**Fig. 2:**
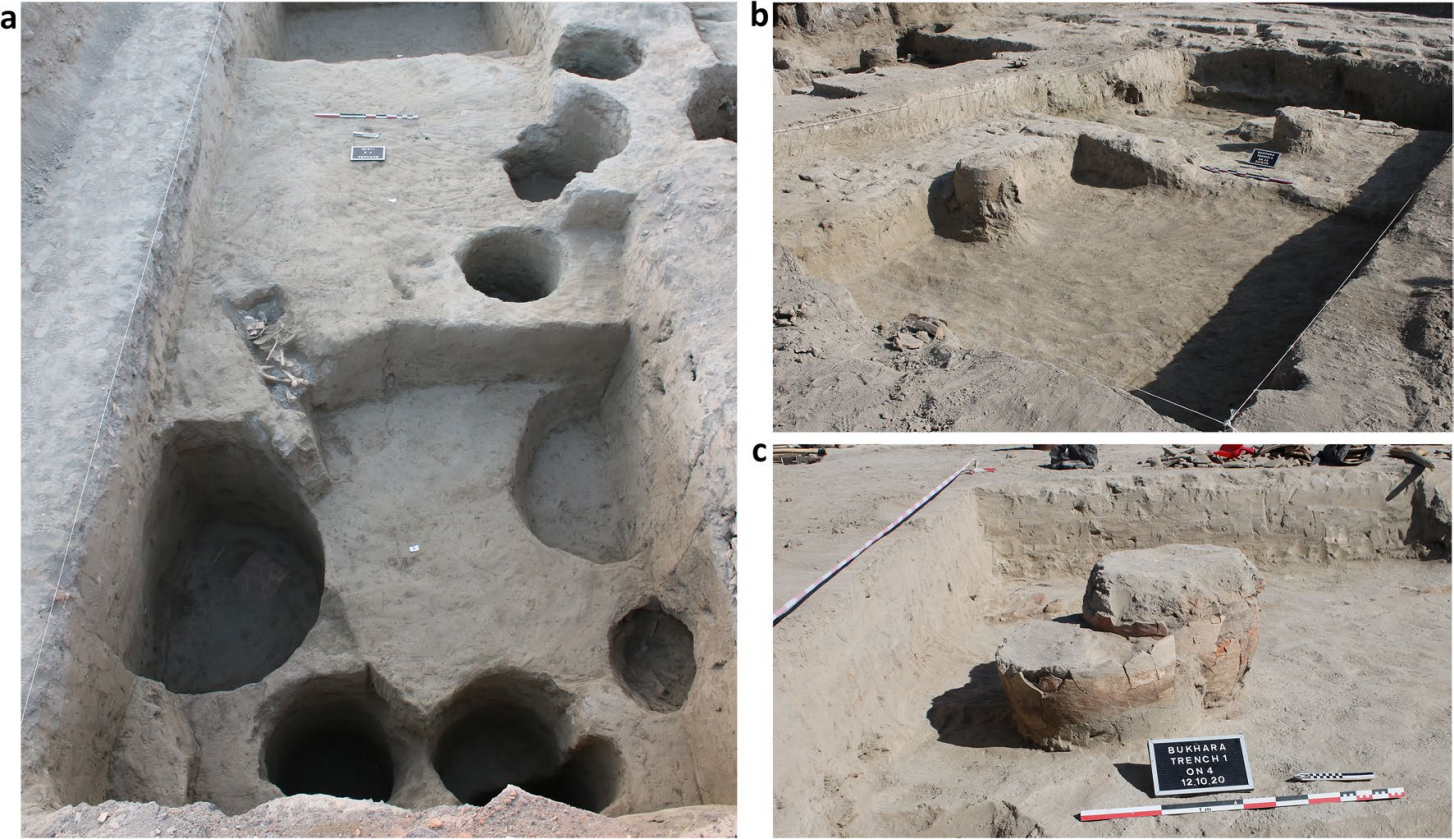
**a** *Badrabs* from Trench 2, partially excavated; **b** *tashnaus* from Trench 4; **c** *tanur* ON4

Since the fall of 2020, the UzAmEB — a collaboration between the Samarkand Institute of Archaeology under the Agency of Cultural Heritage of the Republic of Uzbekistan and the Institute for the Study of the Ancient World at New York University — has been conducting systematic excavations at ca. 0.75 ha large area immediately to the north of the city’s Congregational Mosque and the early medieval city’s main east–west thoroughfare (present-day Nurobod Street), and to the east of the city’s citadel (Fig. 1d). One of the most important results of three extensive seasons of fieldwork (2020, 2021, and 2022) was the identification of the sixth–ninth centuries AD western wall of the inner city (the so-called *shahristan* or *madinah* of medieval Persian and Arabic-language sources) just to the north of the former Banu Asad gate (the main western gate of the city) mentioned in the extant version of Narshakhi’s tenth century AD “*History of Bukhara*” (Tārīkh-i Bukhārā 1984, p. 76). In the area immediately outside this stretch of the inner-city wall, excavations conducted in 2020 and 2021 revealed close to forty wells filled with refuce (*badrab*, Fig. 2a), cesspits (*tashnau*, Fig. 2b), and *tanur*-ovens (Fig. 2c) dating to the tenth through eleventh centuries AD, while at the same time, only very few traces of permanent buildings of the same period were uncovered. Thus, in all likelihood, during the Samanid and early Qarakhanid periods, the area just outside of the city wall and just north of the main western gates housed a small non-permanent basar of small shopkeepers and mobile salesman catering to travelers entering or leaving the city. The refuse-filled wells (*badrab*) and cesspits (*tashnau*) are a phenomenon characteristic of tenth–early thirteenth centuries cities all across Central Asia (Anarbaev 1981). The *badrabs* excavated by the UzAmEB in Bukhara since 2020 are usually round-shaped simple pits with diameters ranging between 70 and 130 cm. Many of them were dug deeper than 7 m into the ground, thus deeper than the present-day water table. *Tashnaus* can reach a similar depth but are usually of a slightly smaller diameter; they are either lined by fired bricks or contain upside-down large storage vessels whose bases have been broken off. Both *badrabs* and *tashnaus* were filed with sediments enriched with botanical and entomological remains, as well as with an enormous amount of ceramic and glass fragments, metal objects (such as coins), and (to a lesser degree) animal bones. Abundant diagnostic ceramic fragments and occasional coin finds from these *badrabs* and *tashnaus* allow us to reliably date them between the tenth and eleventh centuries AD. In addition, the area housed several *tanur*-ovens. They consist of large clay vessels (usually reused storage vessels), dug into the ground in order to save energy and retain heat. They are more difficult to date by themselves, but their stratigraphical context suggests Qarakhanid (eleventh–twelfth centuries AD) period for most of them. By the second half of the twelfth century, this area was, in all likelihood, no longer used as a mobile basar, because at that time, the Banu Asad Gate had been laid down and the city’s new Congregational Mosque had been built in the area just to the south (Tārīkh-i Bukhārā 1984, p. 71); at the same time, the area further to the north had turned into a huge cemetery (Tārīkh-i Bukhārā 1984, p. 76 and ongoing excavations by the UzAmEB).

During the field seasons of 2020 and 2021, we collected a total of forty sediment samples from the UzAmEB excavations (Fig. 1d). Badrab and tashnau contexts were represented by 18 samples (2243.5 l of unprocessed sediment) and non-*badrab/tashnau* contexts (*tanurs* and other contexts) by 22 samples (430.5 l) (for more context details, see Online source 1). The archaeobotanical samples were taken from contexts that appeared to be rich in charred and mineralized material, such as ashy deposits, hearth features, garbage wells, and cesspits. Sediment samples ranged in volume from 5.0 to 426 l; in total, 2674.5 l of anthropogenic sediment were floated and analyzed. Our samples varied in volume, because of availability of the sediments suitable to sampling. Some small features did not always contain 10 l, so we took as much sediment as was available. Although, if contexts were larger, we collected at least 30 l; however, for wells (*badrabs*) and cesspits (*tashnau*), we sampled as much sediment as we could process.

All materials were obtained by water flotation, using an overflow tank system, and air-dried in the shade in the cotton cloth avoiding contamination and sweating, which damages charred materials. Heavy fraction portions of the sediments were collected down to 2.0 mm and light fractions down to 0.355 mm. All heavy fractions were carefully sorted on site for the presence of charcoal, bones, or cultural artefacts, such as ceramic fragments, beads, and glass. All organic materials recovered in the heavy fractions and light fractions were shipped to Germany, while all cultural artefacts were returned back to local collaborators. Light fractions passed through a series of sieves with mesh sizes of 2.00, 1.40, 1.00, and 0.50 mm in the lab. The archaeobotanical remains were sorted, classified, and identified in the Paleoethnobotany Laboratory at the Max Planck Institute for the Science of Human History. Fruits, seeds, and other diagnostic plant remains (other than wood) were identified under a low magnification microscope, a Leica M205C. Length, width, and thickness measurements were made digitally with a Keyence VHX 6000 microscope for all whole wheat, barley, and rice grains. In addition to the grain length, width, thickness, and scutellum lengths were measured for millets that were not enclosed in their paleo and lemma (Table 1). Minimum Number of Individual (MNI) estimates were attempted for barley and wheat grains, where three fragments were counted as one whole grain. The MNI method was not applied to nutshell fragments or wild seeds. Highly fragmentary pieces of grains and legumes were placed in the categories: Cerealia and legume. Cerealia, legume, crop by-products (like rachises, culm nodes, and grape pedicels), and unidentifiable seed fragments were not counted in the totals but are presented in the table in SI 1.

**Table 1:** Average seed measurements for the most prominent grain crops (Online source 3)

	Total	Density	Measured	Not measurable	Average length (mm)	Average width (mm)	Average thickness (mm)	Scutellum length (mm)
**Charred**								
Barley	375	0.1	145	230	5.31	2.94	2.37	
Wheat	254	0.09	101	153	4.21	2.82	2.37	
Broomcorn millet	220	0.08	47	173	2.00	1.78	1.49	1.70
Foxtail millet	339	1.13	84	255	1.65	1.57	1.30	1.07
**Mineralized**								
Barley	4	0.001	1	3	6.75	3.36	2.49	
Wheat	9	0.003	3	6	5.39	3.64	2.49	
Rice	295	0.11	104	191	5.54	2.33	3.29	

Two samples of cotton (*Gossypium* sp.) — Trench 1 (from the same context), mung bean (*Vigna* cf. *radiata*) — Trench 4, and grape (*Vitis vinifera*) — Trench 4 (*badrab*) seeds were selected for AMS^14^C dating and directly dated (AMS) at the Woods Hole Oceanographic Institution Radiocarbon Laboratory, SUERC Radiocarbon Dating Laboratory, and FTMC Vilnius Radiocarbon. All results were calibrated using OxCal v4.4.2 software (Bronk Ramsey 2009, 2020) and the IntCal 20 curve (Reimer et al. 2020).

## Results

### AMS dating

**Fig. 3:**
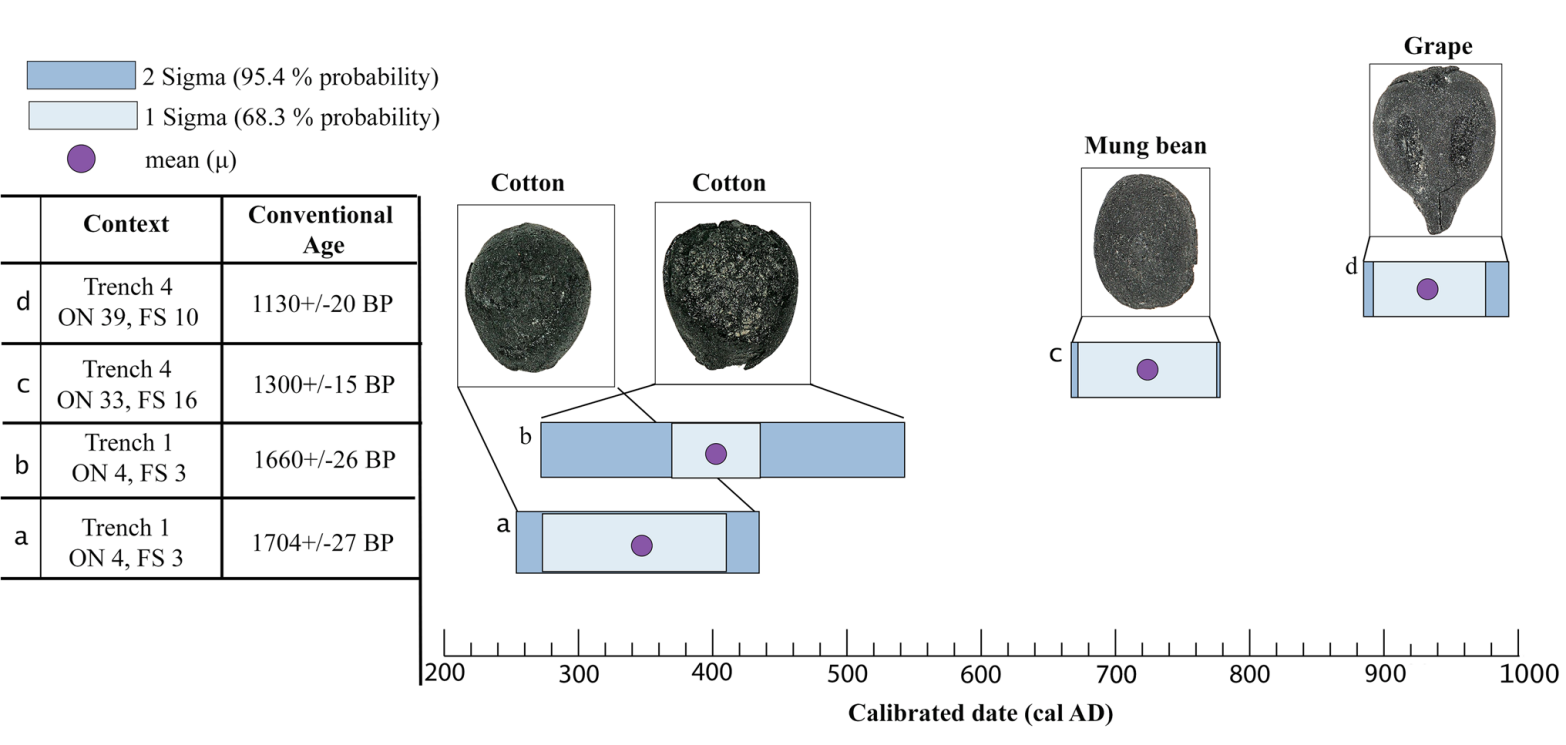
Radiocarbon dates obtained on carbonized grains recovered from Trenches 1 and 4

The results of radiocarbon dating of macrobotanical remains are shown in Fig. 3. Based on 2-sigma calibration, one cotton seed recovered from a *tanur*-oven in Trench 1 (ON-4) with a repurposed large storage ceramic vessel, dated between cal. AD 261 and 532 (SUERC-100308), with a mean at cal. AD 401. Another cotton seed from the same context dated between cal. AD 255 and 416 (FTMC-RS71-1), with a mean at cal. AD 348. Judging from the stratigraphic context of the tanur in question (eleventh–twelfth centuries AD), both seeds predate the *tanur* by at least some 600 years and, consequently, must have been re-deposited here as a result of the Qarakhanid period levelling and construction activities. The same is true for the mung bean from *tanur* ON-33 in Trench 4. This *tanur* also dates to the Qarakhanid period, while the bean itself dated between cal. AD 663 and 775 (OS-165287), with a mean of cal. AD 723. Only the AMS date of a grape pip, recovered from *Badrab* ON-19 in Trench 4, of cal. AD 882–991 (OS-165286), with a mean value of cal. AD 930, roughly corresponds with the conventional date of the stratigraphic context and the diagnostic ceramics from the fill of the *badrab*. Thus, it seems that material from the *tanur*-ovens might potentially be much older than the actual stratigraphic contexts. However, the case of the *badrabs* and *tashnaus* is different: Sample OS-165286 confirms the chronologically homogenous character of the *badrab* and *tashnau* fills, as evident already from the diagnostic ceramics and coins that they contained. This suggests that these waste and drainage pits were backfilled over a relatively short period of time during the Samanid and early Qarakhanid periods.

### Archaeobotany

**Fig. 4:**
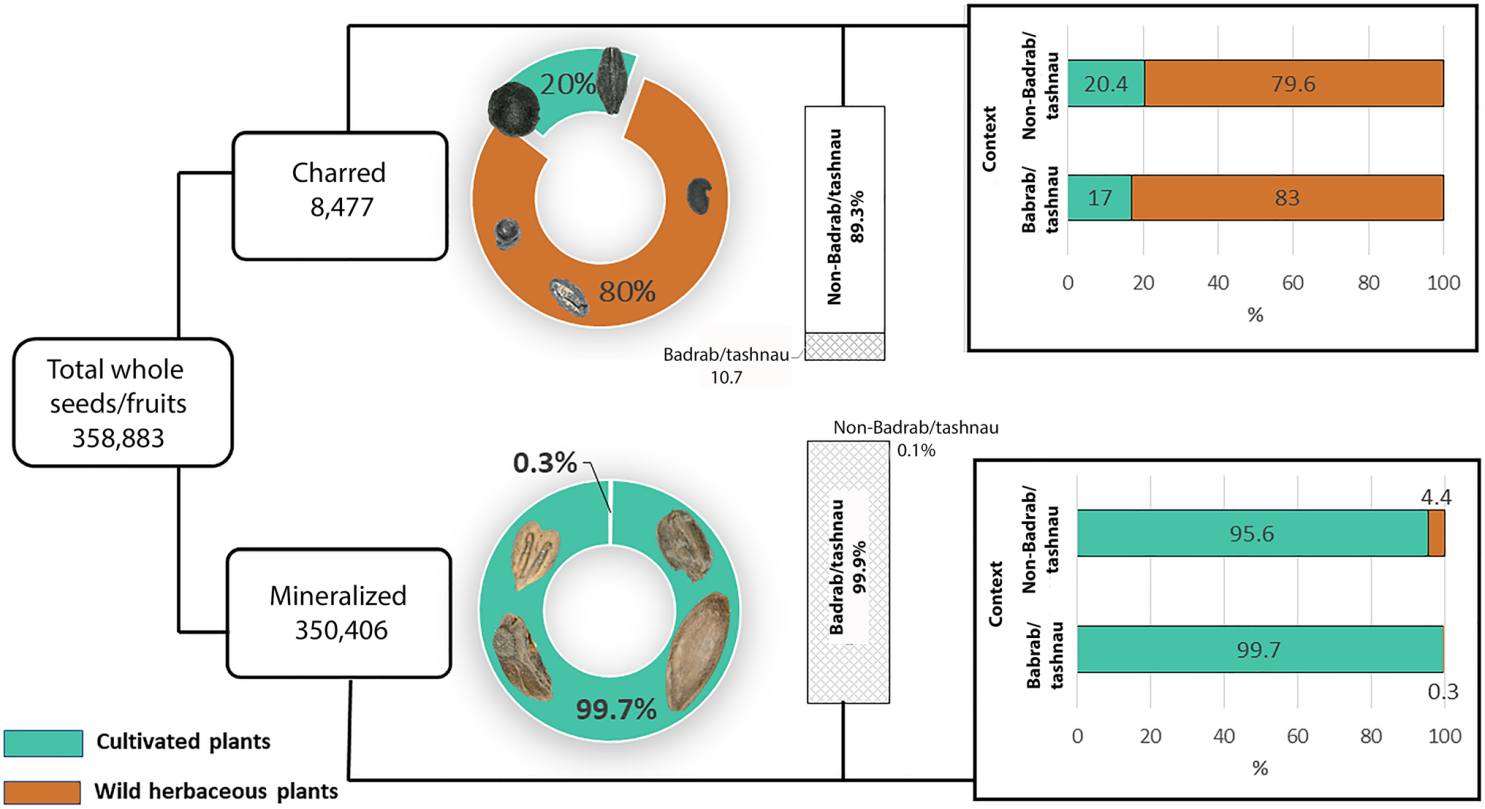
Relative abundances of mineralized and carbonized plant remains in the Bukhara assemblage

A total of 358,883 identifiable seeds and fruit parts have been recovered (Online source 2). The results are clearly framed within different preservation rates. The archaeobotanical assemblage from Bukhara consists of 8477 (3.2 seeds per liter of sediment) charred and 350,406 (131 seeds per liter of sediment) mineralized seeds and fruit remains. The charred specimens were primarily recovered from anthropogenic sediments and the mineralized material was largely recovered from *badrabs* and *tashnaus* dated to the tenth century AD (Table 2). In addition to the identifiable plant remains, there are 42,478 unidentifiable seed fragments. Charred specimens mainly consist of wild plants, while mineralized plants mostly include cultivated crops, fruits, and spices (Fig. 4).

**Table 2:** Economic plants recovered in charred and mineralized modes in Bukhara; *AP*, absence/presence; *U*, ubiquity of seeds from each period; * rare (one-two specimens), ✓ — present (< 1 specimen per liter), ✓✓ — common (> 1specimen per liter), and ✓✓✓ — abundant (> 10 items per liter). Five samples were not included in this table because four samples (FSB 2, FSB 20, FSB 3–21, and FSB 14–21) do not have conventional dates, and one sample (FSB 23) is dated to the third century BC until the first century AD

	4th–eighth centuries AD (4 samples, 201 l)				10th–eleventh centuries AD (28 samples, 2297 l)				14th–fifteenth centuries AD (3 samples, 8.5 l)			
	Charred		Mineralized		Charred		Mineralized		Charred		Mineralized	
	AP	U	AP	U	AP	U	AP	U	AP	U	AP	U
Wheat	✓	0.50	-	-	✓	0.42	✓	0.1	*	0.66	-	-
Barley	✓✓	1	-	-	✓	0.46	*	0.03	*	0.33	-	-
Rice	-	-	-	-	-	-	✓	0.32	-	-	-	-
Broomcorn millet	✓	0.75	-	-	✓	0.42	✓✓	0.25	-	-	-	-
Foxtail millet	✓	0.5	-	-	✓✓	0.46	-	-	-	-	-	-
Lentil	✓	0.25	-	-	✓	0.14	✓	0.36	-	-	-	-
Pea	-	-	-	-	✓	0.1	*	0.03	-	-	-	-
Chickpea	✓	0.25	-	-	-	-	-	-	*	0.33	-	-
Mung bean	✓	0.25	-	-	-	-	-	-	-	-	-	-
Eggplant	-	-	-	-	-	-	✓	0.1	-	-	-	-
Cotton	✓	0.75	-	-	✓	0.21	✓✓	0.39	✓	0.33	-	-
Flax	-	-	-	-	-	-	✓	0.1	-	-	-	-
Coriander	-	-	-	-	*	0.03	✓	0.28	-	-	-	-
Cress	-	-	-	-	-	-	✓	0.17	-	-	-	-
Sesame	-	-	-	-	-	-	*	0.03	-	-	-	-
Pepper	-	-	-	-	-	-	*	0.03	-	-	-	-
Cumin	-	-	-	-	-	-	✓	0.07	-	-	-	-
Sumac	-	-	-	-	-	-	✓✓	0.32	-	-	-	-
Melon	-	-	-	-	-	-	✓✓	0.57	-	-	-	-
Watermelon	-	-	-	-	-	-	✓	0.39	-	-	-	-
Russian olive	-	-	-	-	-	-	✓✓	0.53	-	-	-	-
Walnut	-	-	-	-	*	0.03	-	-	-	-	-	-
Fig	-	-	-	-	-	-	✓✓✓	0.5	-	-	-	-
Mulberry	-	-	*	0.25	-	-	✓✓	0.57	-	-	-	-
Pomegranate	-	-	-	-	-	-	✓✓	0.57	-	-	-	-
Apple	*	0.25	-	-	-	-	✓	0.53	-	-	-	-
Pear	-	-	-	-	-	-	*	0.07	-	-	-	-
Grape	✓	0.50	-	-	✓	0.46	✓✓✓	0.6	-	-	-	-
Peach	-	-	-	-	*	0.03	-	-	-	-	-	-
Prunus sp.	-	-	-	-	-	-	✓	0.07	-	-	-	-

### Charred material

Nearly 90% of the charred plant remains were recovered from ashy deposits, *tanur* fillings, and hearths (Fig. 4), while the remaining 10% represent charred specimens recovered from *badrabs* and *tashnaus*. Specimens recovered from non-*badrab/tashnau* deposits consist of 20.4% cultivated crops (including fruits/nuts) and 79.6% wild herbaceous plants, while charred plant materials coming from *badrabs* and *tashnaus* are predominantly wild herbaceous plants (83%).

#### Charred — non-*badrab/tashnau* deposits

The Bukhara archaeobotanical samples from the non-*badrab/tashnau* deposits yielded a large spectrum of grain crops, representing the dominant category of recovered cultivated plants, including barley (*Hordeum vulgare*) (*n* = 336), foxtail millet (*Setaria italica*) (*n* = 323), wheat (*Triticum aestivum*) (*n* = 214), and broomcorn millet (*Panicum miliaceum*) (*n* = 210). Charred crop-processing by-products, notably hexaploid wheat, hulled barley, and *Aegilops tauschii* rachises, wheat (*Triticum* sp.) spikelet forks, and culm nodes were recovered only from 5 out of 22 samples, likely because grains were brought to residential areas post-processed and chaff would have been removed elsewhere (see discussions regarding this reasoning in Stevens 2003). The most ubiquitous charred legume in the non-*badrab/tashnau* contexts is lentil (*Lens culinaris*) (*n* = 41), while the most numerous is mung bean (tentative ID) (*Vigna* cf. *radiata*) (*n* = 128), which was recovered from only one sample. Pea (*Pisum sativum*) (*n* = 4) and chickpea (*Cicer arientinum*, *n* = 2) are the least abundant cultivated legumes identified in the assemblage (Table 2). Another abundant economic plant was cotton (*Gossypium* sp., *n* = 199). The category — fruits and nuts — comprises all of the fleshy fruiting species and fragments of nutshells; overall, this is a small proportion (6.1%) of the charred economic plants. The fleshy fruits include grape (*Vitis vinifera*) pips (*n* = 71) retrieved from the non-*badrab/tashnau* deposits. In addition to the grape seeds, one apple/pear seeds (*Malus/Pyrus*) and one stone fragment of peach (*Prunus persica*) were recovered.

The charred seed assemblage from the non-*badrab/tashnau* context consists of 79.6% wild seeds (Fig. 3), representing more than 50 taxa. The majority of the wild plant remains cannot be assigned to a species level and were only identified to the family or genus level. The most ubiquitous and numerous types are Amaranthaceae (*n* = 1279), *Chenopodium* sp. (*n* = 326), Fabaceae (*n* = 680), *Medicago/Melilotus* spp. (*n* = 647), *Alhagi* sp. (*n* = 222), Panicoid (*n* = 286), and *Panicum* sp. (*n* = 253).

#### Charred — *badrab/tashnau* deposits

Charred plant remains recovered in *badrabs* and *tashnaus* are represented only by 17% of cultivated plant remains, such as wheat (*n* = 40) and barley (*n* = 39). Only 10 broomcorn and 16 foxtail millet grains were recovered from *badrabs* and *tashnaus* in a carbonized state. The extreme disproportion may be the result of different seeds having different likelihoods of becoming mineralized. Crop processing by-products or chaff consisted of barley rachises (*n* = 5), grape pedicels (*n* = 2), and culm nodes (*n* = 6). We recovered only 1 charred lentil, 2 peas, and 9 cotton seeds in the *badrabs* and *tashnaus* of Trench 4.

**Fig. 5:**
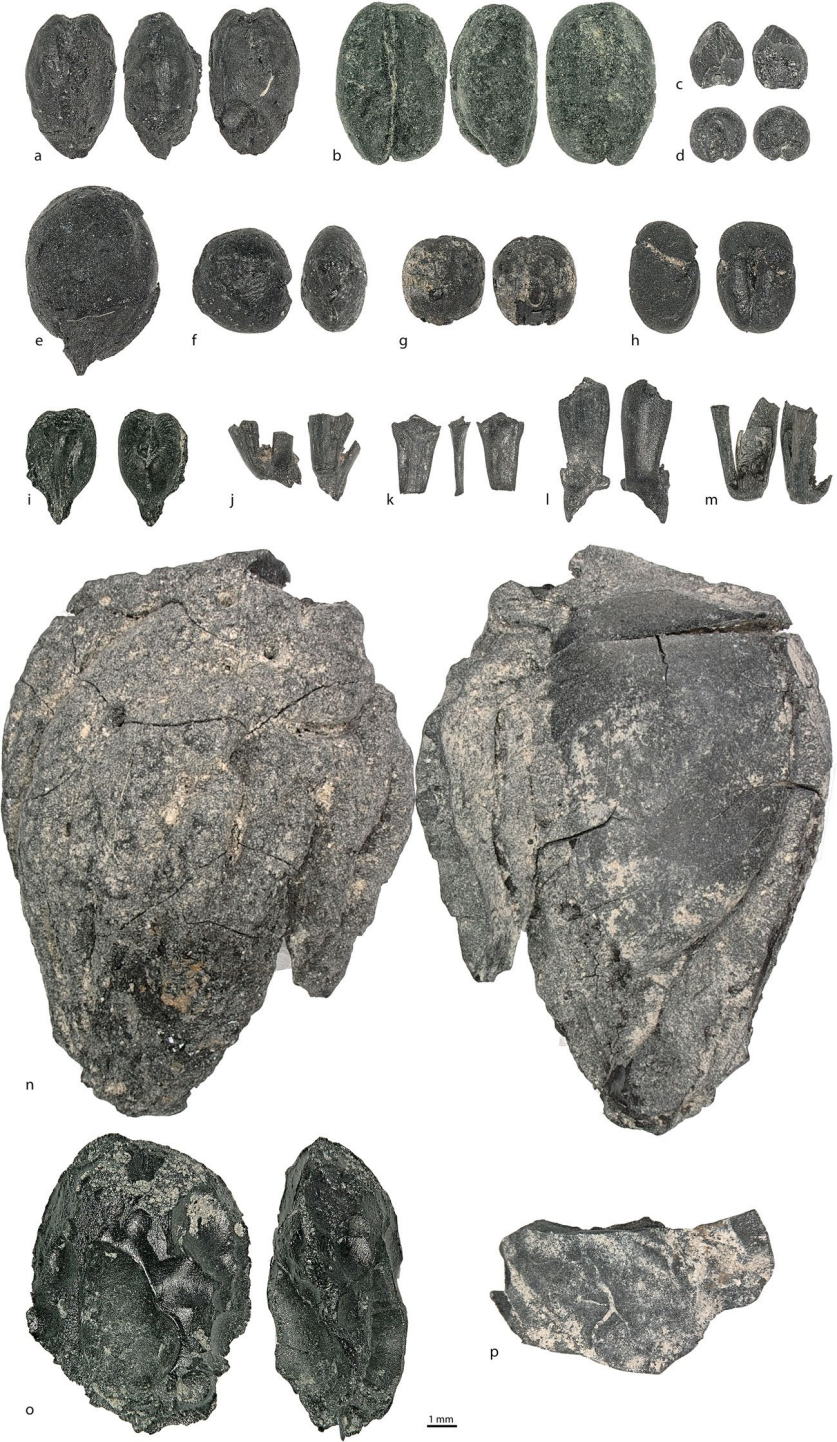
**a** *Hordeum vulgare* (grain); **b** *Triticum aestivum* (grain); **c** *Panicum miliaceum*; **d** *Setaria italica*; **e** *Gossypium* sp.; **f** *Lens culinaris*; **g** *Pisum sativum*; **h** *Vigna* cf. *radiata*; **i** *Vitis vinifera* (pip); **j** *Triticum* cf. *spelta* (spikelet); **k** *Hordeum vulgare* (rachis); **l** *Triticum aestivum* (rachis);** m** *Aegilops tauschii* (spikelet); **n** *Prunus* cf. *persica*; **o** *Prunus* sp. (nut exocarp); and **p** *Juglans regia*

In addition to staple crops, a low number of charred fruit and nut remains was recovered in the *badrabs* and *tashnaus*, notably a single melon seed (*Citrullus lanatus*), a Russian olive seed (*Elaeagnus angustifolia*), shell fragments of walnut (*Juglans regia*, *n* = 2), full grape berries (*n* = 7), grape pips (*n* = 18), a peach stone (*Prunus persica*), and one fruit exocarp likely *Prunus* sp. (Fig. 5o). There were 35 charred wild herbaceous taxa in the cesspit.

### Mineralized material

Nearly 99.7% of the mineralized specimens are from economically significant plants, and they mainly come from *badrab* or *tashnau* deposits. There are only 67 mineralized seed remains recovered from non-*badrab/tashnau* deposits, where three seeds belong to cultivated plants and the rest to wild plants within two taxa: *Lithospermum arvense* and *Convolvulus* sp.; *L. arvense* is commonly preserved in a mineralized state regardless of the context, due to the biomineralized nature of these botanical remains (Pustovoytov and Riehl 2006; Messager et al. 2010).

**Fig. 6:**
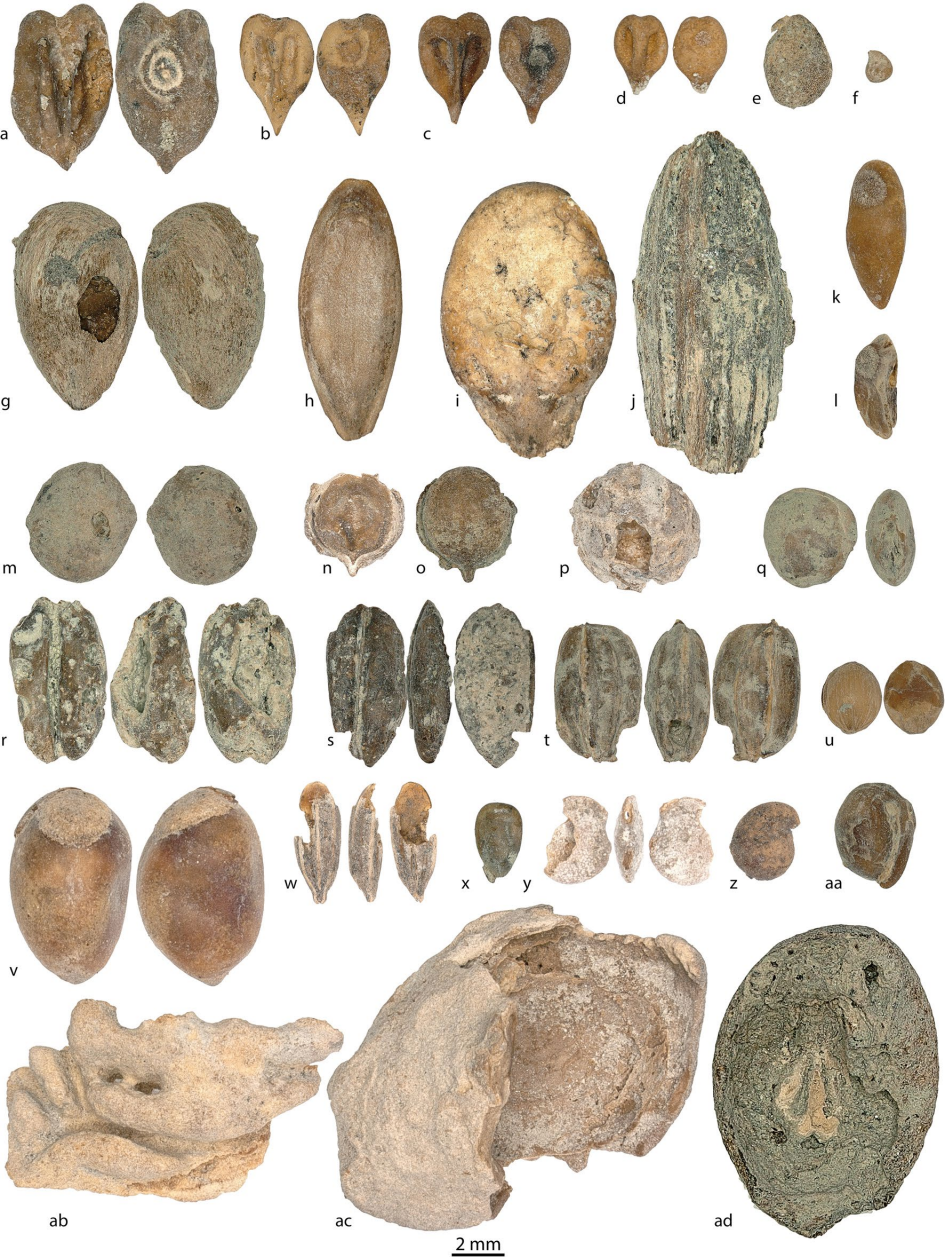
**a**, **b**, **c**, **d** *Vitis vinifera*, illustrating the range of diversity;** e** *Morus nigra/M. alba*; **f** *Ficus carica*; **g** *Malus domestica*; **h** *Cucumis melo*; **i** *Citrullus lanatus*; **j** *Elaeagnus angustifolia*; **k**, **l** *Punica granatum*; **m** *Rhus coriaria*; **n**, **o** *Coriandrum sativum*; **p** *Piper nigrum*; **q** *Lens culinaris*; **r** *Triticum aestivum*; **s** *Hordeum vulgare*; **t** *Oryza sativa*; **u** *Panicum miliaceum*; **v** *Gossypium* sp.; **w** cf. *Cuminum cyminum*; **x** *Sesamum indicum*; **y** *Solanum* cf. *melongena*; **z** *Capparis spinosa*; **aa** no ID-seed D; **ab** *Prunus persica*; **ac** *Prunus* cf. *cerasifera*; and **ad** grape berry

The *badrabs* and *tashnaus* provided a high relative abundance (99.7%) and high density (131.2 seeds per liter) of cultivated plant remains. The presence of cultivated fruits is notable, especially grape pips (*n* = 241,366), which were recovered in all *badrab* and *tashnau* samples, in several samples (7 out 18) accompanied by their berries and pedicels. In addition to grapes, mulberries (*Morus* sp.) (*n* = 14,178), figs (*Ficus carica*) (*n* = 52,795), pomegranates (*Punica granatum*) (*n* = 27,324), Russian olives (*Elaeagnus angustifolia*) (*n* = 2710), melons (*Cucumis melo*) (*n* = 3216), watermelons (*Citrullus lanatus*) (*n* = 67), apples (*Malus domestica*, *n* = 2206), and apple/pear (*Malus/Pyrus*) (*n* = 160) (Fig. 6) are all abundant plant remains from the *badrab/tashnau* deposits.

Although there was high abundance and density of seeds from fleshy fruits, principally grapes, figs, apples, melons, pomegranates, and Russian olives; cereals and legumes were also recovered. In particular, broomcorn millet (*n* = 318) is the most common mineralized cereal followed by rice (*Oryza sativa*) (*n* = 295). Wheat (*n* = 9) and barley (*n* = 4) were less well represented in the *badrabs* and *tashnaus*. Lentils (*n* = 189) are the most numerous and ubiquitous legume. The oilseed and fiber crops of flax (*Linum* cf. *usitatissimum*, *n* = 14) and cotton (*n* = 412) were identified in the assemblage. In addition to the most common fruits and annual crops, we recovered possible eggplant seeds (*Solanum* cf. *melongena*, *n* = 31).

Among the recovered economic plant remains, it is important to note that the first solid evidence in Central Asia of sumac (*Rhus coriaria*) (*n* = 992), coriander (*Coriandrum sativum*) (*n* = 62), black pepper (*Piper nigrum*) (*n* = 1), sesame (*Sesamum indicum*) (*n* = 1), and likely cumin (*Cuminum cyminum*, *n* = 5) all come from the Bukhara assemblage. Wild plant seeds are rare in the *badrabs* and *tashnaus*; nevertheless, 25 taxa were recovered in this study. The most abundant wild seeds are *Portulaca oleracea* (possible a minor crop), *Convolvulus* sp., and *Lithospermum arvense*. Some taxa appear in just one sample, suggesting an accidental entry with wind (e.g., Asteraceae, *Carex* sp., *Cladium mariscus*, *Papaver* sp., *Polygonum* sp., and *P. ariculare*).

## Discussion

### Preservation biases

The Bukhara assemblage provides an opportunity to understand human–plant relationships across the occupation period of an urban center. However, it is important to consider preservation biases; samples taken from hearths, ash lenses, and midden deposits most often consist of charred plant remains like cereal crops, legumes, chaff, and synanthropic plants representing food preparation, crop processing activities, and dung burning (Renfrew 1973). Carbonization/charring is the most common mode of archaeobotanical preservation, but certain plants do not survive carbonization/charring or being exposed to charring during processing/consumption (van der Veen 2007). Therefore, the Bukharan *badrabs* and *tashnaus* provide a rare opportunity to see what crops are not being identified in assemblages that lack a mineralized component.

Mineralization often relates to calcium carbonate or calcium phosphate replacing carbon, leaving a fossil of the botanical material (Marshall et al. 2008). Excrement and food residues can increase phosphate levels within a *badrab*. Preservation can be facilitated by the continued presence of water or rapid accumulation of waste during the mineralization process, leading to an anaerobic environment. In such a context, ions can defuse and become concentrated, resulting in calcium phosphate precipitate, which often appears within urban occupation deposits (Marshall et al. 2008; Murphy 2014). Additionally, there are a few mineralized specimens in the assemblages outside the *badrabs* and *tashnaus*, specifically from plants that produce especially dense testa or pericarp, facilitating the biomineralization processes (e.g., *Lithospermum arvense*) (Pustovoytov and Riehl 2006; Messager et al. 2010).

It is not surprising that the data from the middens or ash deposits looks very different from that of the *badrabs* and *tashnau*s, given the different conditions facilitating and deposition preservation. *Badrabs* and *tashnaus* accumulate consumption refuse, kitchen by-products (Hondelink and Schepers 2020), or domestic rubbish (Smith 2013). It is quite common to recover grape pips and fig achenes in the *badrab* and *tashnau* deposits; in the same way, mulberry and apple seeds could have become incorporated into the *badrabs* and *tashnaus* in Bukhara within human feces. Moreover, Murphy (2014) reported that millets are also frequently recovered in a mineralized state, since some millet grains will retain their indigestible hull and small size; these specimens pass through the gastrointestinal tract. Correlatively, only millet grains with articulated husks were recovered from the Bukharan *badrabs* and *tashnaus*, possibly suggesting that a few grains retained their palea and lemma after processing and were inadvertently consumed with de-hulled grain porridge. Kitchen by-products mostly consist of large inedible parts of fruits, nuts, and vegetative parts (e.g., chaff) that enter the *badrab* or *tashnau* (cesspit) with general rubbish disposal (Greig 1982). In addition, cereals are only present in low abundances. The presence of legumes in the Bukhara *badrabs* and *tashnaus* is likely the result of disposal of kitchen waste. Moreover, whole grains of cereals and pulses are rarely encountered in cesspit deposits (Smith 2013), since protein-rich pulses (e.g., lentils) would normally have been digested completely (Hondelink and Schepers 2020). Therefore, we suggest that the presence of lentils in the *badrabs* and *tashnaus* illustrates that they were deposited with kitchen waste as they could not have survived digestion. Cultural artefacts like glass, ceramic wares, and metal object recovered from the *badrabs* and *tashnaus* and archaeobotanical data illustrate a multifunctional role for these pits in Bukhara.

Wild and uncultivated plant remains (e.g., *Portulaca oleracea*, *Chenopodium* sp., *Carex* spp., *Convolvulus* sp., *Galium* spp., *Polygonum* spp., *Polygonum ariculare*, wild *Setaria*, Caryophyllaceae, and Solanaceae) were recovered in the *badrab* deposits as well, and may or may not represent wild plant foods. Some of these plants, such as *Portulaca*, *Chenopodium*, and many species of Solanaceae, have been cultivated as minor crops in Eurasia, and have also been foraged from the wild (van der Veen et al. 2011; Danin et al. 2014; Gao 2021). They could have been introduced to the pit with cereal chaff or grass floor matting, as kitchen waste/rubbish disposal, or they may have grown around the pits, entering through seed rain (Moffet 1992; Smith 2013). The presence of charred grains in the *badrab* and *tashnau* samples may indicate the disposal of burnt kitchen waste. Some scholars have also proposed the possibility that ash was used as a cleaning additive to cesspits (Smith 2013) or as disinfectant (Murphy 2014). Nevertheless, some of the charred taxa recovered in the non-*badrab/tashnau* deposits (e.g., *Chenopodium* sp., *Medicago* sp., and *Alhagi* sp.) are potential indicators of dung burning, and many of them are from weedy herbaceous species (see discussions regarding this reasoning in Miller 1984).

The fact that arboreal crops, such as figs, pomegranates, and mulberries, are less likely to be recovered from a charred archaeobotanical assemblage suggests that comparison with the pre-Islamic data recovered from the *tanur*-oven and data dated to Islamic period coming from the *badrab/tashnau* deposits in this study is problematic. Variations in the quantities of specific species may be more likely to reflect their likelihood of preservation and carbonization(charring)/mineralization than their economic significance. Some plants are consumed before they start seed production (e.g., cucumber), others are less likely to enter a fire, due to the method of cooking (e.g., grinding, pounding, or heat treatment), some cultural practices, such as parching, increase the likelihood of a seed entering the archaeobotanical record, some species produce seeds that do not survive carbonization/charring, and digestion and mastication may result in plant remains for certain species not preserving (e.g., legumes) (Wright 2003; Hondelink and Schepers 2020).

### Fruits and nuts

Archaeobotanical research in Central Asia has focused on field crops, with far less attention paid to long-generation perennials. Historical and archaeological evidence illustrate the economic importance of fruit and nut cultivation and trade across the region going back at least two millennia (Spengler 2019a). The accounts on Alexander’s conquest of Sogdiana mention hunting parks and lavish gardens between Samarkand and Panjakent (Diod. XVIII, prol. 2, 26; Curt. VIII 1, 10–19, Ešonkulov 2004), and historians mention the cultivation of apricots (*Prunus armeniaca*), peaches, apples, pears (*Pyrus communis*), cherries/plums (*Prunus* sp.), quinces (*Cydonia oblonga*), plums (*Prunus* sp*.*), pomegranates, figs, almonds (*Prunus dulcis*), and walnuts on the Central Asian oases (Barisitz 2017, p. 80). In the *History of Bukhara*, it was reported that Bukhara was filled with courts, gardens, and parks; for example, within one of the courts, extending from Rigistan to Dashtak, there were four beautiful gardens where pears, almonds, hazelnuts (*Corylu*s sp.), cherries, and grapes were grown (Narshakhi 1954). For example, the Qarakhanid rulers invested in Persianate gardens (e.g., Shams al-Mulk) (Karev 2013). Gardens were likely for elite use; therefore, it is almost certain that there were also many orchards around Bukhara and its suburbs that contribute to the local economies and improvement of human nutrition. Ibn Ḥawqal (977, p. 249) wrote, “Fruits of Bokhara are more excellent than the fruits of any part of Maweralnahr.”

Grape seeds were the most numerous perennial crop in the *badrabs*. Grapes are also the most versatile of the perennials, as they can be grown in household gardens or in large irrigated fields. Grapes can be used to produce wine, juices, syrups, and raisins. As one ancient example, in Iskijkant, a rural town ca. 27 km north of Bukhara, a special sweet grape syrup was prepared by dipping almonds, and later it was sold in the markets (Narshakhi 1954). There was a wine house (*maikhona* in Uzbek: *mai* — wine, *khona* — house) in Afrasiab, dated to the sixth–seventh centuries AD (Akhmedov 2013), the dimensions of which suggest the mass production of wine. Islamic alcohol prohibition does not appear to have reduced the viticulture industry in this region (Brookshaw 2014). Russian olive stones attached to grape pips likely indicate that they were being processed together, presumably for wine, and historic accounts note that Russian olives can be used to facilitate the fermentation process (Askarov 1977; Mir-Makhamad et al. 2021).

The most widespread species of mulberry in Central Asia are the black (*Morus nigra*) and white (*M. alba*) mulberries. The white mulberry is native to China, and has been intensively cultivated in China for several millennia (Sharma et al. 2000). When silk production spread to other countries, the range of this mulberry also expanded (Vijayan et al. 2011). The white mulberry was historically cultivated for sericulture (Bretschneider 1935), to feed domesticated silkworms (*Bombyx mori*). The black mulberry is thought to have originated in southwest Asia (Wiersema and Leon 1999) and is widely cultivated for its fruit (Tutin 1993). Prior to this study, only Bubnova (1987) reported having found ancient mulberry seeds in Central Asia; she claimed that they were recovered among other cultivated fruits at the Bazar-Dara site, a high-elevation medieval silver-mining town (eleventh century AD, Tajikistan). However, historically in Central and southwest Asia, mulberries have been highly appreciated not only in sericulture but also for their sweet fruits, consumed fresh or as jam (Sánchez 2002). Both species are cultivated in orchards or planted as property markers and only loosely maintained; the trees are grown along street in small “*kishlaks*” (villages) in Tajikistan and Uzbekistan fruits of both species are consumed by local people.

The third most numerous category of fruit seeds is the fig. The native area of the common fig is unknown, as they were widely dispersed in prehistory (Dickson and Dickson 1996), including, reportedly, into Central Asia (Mars 2003). It is likely that it spread out of southwest Asia during the fourth millennium BC (Kislev et al. 2006; Fuller and Stevens 2019). The earliest archaeobotanical remains of *Ficus carica* in China date back more than a millennium (Jiang et al. 2013). Our study provides the earliest archaeobotanical evidence of figs in Central Asia in the tenth century AD; figs were also mentioned only in the Bazar-Dara study from the cultural deposits dated to the eleventh century AD (Bubnova 1987). From contexts dating to a few centuries later (thirteenth to fifteenth centuries AD), fig remains are the most common imported fruit recovered in the capital of the Mongolian Empire, Karakorum (Rösch et al. 2005).

Like figs and mulberries, pomegranate seeds are not found in a carbonized/charred state in Central Asia, complicating discussions of their earliest spread into the region. Being drought tolerant, the plant is extensively cultivated in Uzbekistan today, where a frost-resistant variety evolved (Levin 2006). While scholars have claimed that pomegranate cultivation in Central Asia dates back as far as three millennia (Chandra et al. 2010), there really are no data to support such a statement. Starting in the first millennium AD, pomegranate motifs become common on ceramics and wall paintings found on sites along the Silk Road. For example, pomegranate motifs were identified on the mural paintings in Kara-Tepe, Termez (Levin 2006), Afrasiab (mid to the second half of the seventh century AD, Uzbekistan) (Al’baum 1975), as well as on the Afrasiab frescos (Levin 2006), and at Panjakent (sixth–eighth centuries AD, Tajikistan) (Azarpay 1981). The pomegranate motif was stamped in the tableware at Kafir-Kala (seventh–eighth centuries AD), and it was one of the decorative elements of a painted vase (seventh–eight centuries AD) (Compareti 2011) and porcelain dish (likely produced in China) at Afrasiab (twelfth century AD) (Shishkina 1979). In addition, pomegranate peels were reportedly handpicked at the Balalyk-Tepe site (fifth–seventh centuries AD, Uzbekistan) (Al’baum 1960). Pomegranate was an object of artistic expression not only in Central Asia but also across Western Asia (Zadeimanian and Sahragard 2020), in the Mediterranean (Levin 2006), and east to ancient Khotan, where an altar frame with a pomegranate motif on the center was found to date to the fourth century AD and pomegranate motifs on the silk fragments in the ancient city of Gaochang (near Turpan) dated to the eight–eleventh centuries AD (Whitfield and Sims-Williams 2004). The pomegranate not only was an economically important fruit but was also a symbol of fertility, life (Zadeimanian and Sahragard 2020), and abundance (Cammann 1976).

Melon seeds were also prominent in the Bukharan assemblage. The debate over the origin of the sweet melon is still not fully resolved (Wang et al. 2020), although the earliest archaeobotanical evidence comes from eastern China (Jiang et al. 2013). Even the trans-Eurasian dispersal of the crop remains controversial, as Peña-Chocarro and Pérez-Jordà (2019) recently argued that the sweet melon did not arrive in Europe until the fifteenth century AD, claiming that earlier proposed melon seeds were actually from a vegetable melon, similar to the elongated Chante melon. Taking into consideration a wall painting with banqueters, dated to the eighth century AD at Panjakent (Hensellek 2019), it appears that the sweet melon was present in Central Asia already by that time. Unlike melons, the origins of the watermelon are more widely accepted, as genetic studies place the progenitor in West Africa (Chomicki and Renner 2015). Melon and watermelon seeds were recovered together at the Erk-Kala site dating to the first half of the first millennium AD in Turkmenistan (Usmanova 1963), Balalyk-Tepa in the seventh centuries AD in Uzbekistan (Al’baum 1960), Tirmazaktepa (Negmatov et al. 1973) and Bazar-Dara (Bubnova 1987) in the eleventh–twelfth centuries AD in Tajikistan, and in Taraz (Kazakhstan) and Krasnaya Rechka (Kyrgyzstan) dated to the seventh–eighth centuries AD (Baipakov and Goryacheva 1999). The ratio of watermelon to melon in the Bukhara assemblage is 1:35, and watermelon may have been a minor fruit in the region compared to melons.

The apple originated in Central Asia, despite the fact that the seeds are quite rare in early archaeological contexts, with only a few apple seeds from the sites dating to periods prior to the first millennium BC (for more details, see Spengler 2019b). There is more solid evidence for apple consumption from the first millennium AD; for example, Baipakov (1999) reported an entire charred apple from Kuyuk-Mardan (Otrar oasis, Kazakhstan, seventh–eighth centuries AD). Contemporaneous, apple cores were recovered by archaeologists from Mount Mugh in Tajikistan (Vasil’ev 1934). Apple seeds have been recovered from the tenth–eleventh centuries AD settlements of Bazar-Dara (Bubnova 1987), Tashbulak (Spengler et al. 2018), and Paykend (Mir-Makhamad et al. 2021). Apples were more likely to have been imported from the foothills, as they do not grow well in the heat of the desert (Mir-Makhamad et al. 2021).

### Agriculture

First millennium AD Bukhara was surrounded by well-irrigated land, and at least 12 major feeder canals were mentioned by Narshakhi (1954, pp. 44–45) as watering agricultural fields in the region. Although cereal grains occurred in low abundance compared with the number of fruits, grains were undoubtedly important crops in the region since Bukharan wheat was reportedly used as currency prior to the second half of the fifth century AD (1984, p. 49). Rice and millets were the most abundant cereal grains in the Bukharan badrabs and tashnaus, and we believe that rice was cultivated in the Bukhara Oasis and adjacent regions to the Zarafshan River. Despite the fact that millet is often considered a low-income or risk-mitigating crop (Mir-Makhamad et al. 2021), or that it was only cultivated in small towns, homesteads, or by people living in seasonal camps in the first millennium AD in southern Central Asia (Hermes et al. 2018), millet at Bukhara likely represents a signal of the intensification of agricultural production (Miller 2015) or agricultural diversification signal. Moreover, since cereal chaff was rare, we believe it is likely that the people who occupied the Bukhara shakhristan received clean harvested grain or flour and were not involved in cultivation, threshing, or winnowing. The presence of winter and summer crops in Bukhara illustrates a well-established land-management and crop-rotation system. During the Qarakhanid period, land tended to be privately owned and could be bought and sold; larger holdings were likely cultivated by tenants under a sharecropping regime (Paul 2021).

Cotton processing at the site is attested by the abundance of charred cotton seeds recovered from the earliest period (third–fourth centuries AD) at Bukhara. Bukhara and its suburbs were historically an area of clothing production (Tārīkh-i Bukhārā 1984, pp. 18–21, 28–29); notably, cotton clothes from Zandana, a rural town and district ca. 35 km to the northwest of Bukhara, were exported to Iraq, Fars, Kirman, and Hundistan, while Bukharan textiles were exported to Syria, Egypt, and as far west as Rome (Tārīkh-i Bukhārā 1984, p. 28). Currently, the earliest evidence for cotton cultivation outside India dates to the early first millennium AD in Egypt (van der Veen et al. 2011). However, cotton appears in Central Asian archaeological context starting from the fourth century AD in Bukhara, Uzbekistan (our study, directly dated), and Kara-Tepe, Uzbekistan relatively secure based on associated contexts (Brite et al. 2017). Cotton seeds were recovered by archaeologists at the Aktobe 2 settlement along the middle course of the Syr-Darya River (first half of the first millennium AD; Kazakhstan) (Maksimova et al. 1968); however, there are no context details, so follow up work is needed. Among the handpicked remains of cultivated plants recovered from Balalyk-Tepa (fifth–seventh centuries AD; Uzbekistan), cotton bolls were noted in the archaeological report (Al’baum 1960); although in this case too, following up investigations are required. However, cotton seeds and textiles were reported from Mugh (seventh–eight centuries AD; Tajikistan) (Vasil’ev 1934; Danilevsky et al. 1940). Cotton was the most ubiquitous category of seeds at Merv (seventh–twelfth centuries AD; Turkmenistan) (Nesbitt et al. 1994). Its seeds were identified at Paykend from Qarakhanid layers (tenth–twelfth centuries AD, Uzbekistan) (Mir-Makhamad et al. 2021), in Panjakent (seventh–eighth centuries AD, Tajikistan, unpublished), and in Novopokrovka-2 (sixth–seventh centuries AD, Kyrgyzstan, unpublished).

Mung beans likely originated in South Asia (Castillo et al. 2016; Pokharia et al. 2021), and have only been reported from a few Central Asian archaeological sites; therefore, it is possible that the Bukharan mung beans could have been imported from further south. Mung beans were supposedly hand-picked by archaeologists at the Balalyk-Tepe site (seventh century AD, Uzbekistan) (Al’baum 1960). A khum reported as being filled with burned mung beans was recovered at the Minguryuk settlement in Tashkent (eleventh–twelfth centuries AD, Uzbekistan) (Filanovich 1983). They likely spread north through the mountain passes of Swat, as they have been identified in first millennium BC archaeobotanical assemblages there (Spengler et al. 2021), as well as possibly being identified at Wupaer (1189–418 BC) in Xinjiang on the other side of the Kashmir Mountain passes (Yang et al. 2020). Mung beans, rice, and cotton might have spread from South to Central Asia with Sogdians at the beginning of the first millennium AD.

### New tastes and flavors

Spices and condiments, notably black pepper, cumin, coriander, dill (*Anethum graveolens*), chili (*Capsicum* annuum), garlic (*Allium sativum*), cinnamon (*Cinnamomum verum*), and sumac, are the constituent parts of Central Asian cuisine today. Since spices and condiments usually only preserve in a mineralized state (Alonso Martinez 2005), there remains a lack of data regarding their ancient use. The culinary roles of spices are significantly different between northern and southern Central Asia (Anderson et al. 2018). Despite the differences, black pepper is the most common and widespread spice across Central Asia today. Since only one pepper seed has been recovered in this study, we assume it would have been a “luxury” good and avoid further speculation, although pepper was a trade item over the marine routes since the first millennium BC, traveling to northeastern Africa and the Mediterranean, reaching Central Europe already in the first century AD (Reed and Leleković 2019), and China in the second century BC (Spengler 2019a).

The earliest finds of coriander seeds come from sixth and fifth millennia BC sites in Israel and from fourth to second millennia BC sites in Syria, Turkey, and Greece. Zohary and colleagues (2012) proposed that its cultivation could have taken place already by the second millennium BC, when coriander mericarps were found in Egypt, outside their projected wild zones. Coriander was a popular condiment in Roman cuisine (Livarda and van der Veen 2008). It is the most common plant in gardens of Uzbekistan and neighboring countries today, and it is still widely collected from the wild. In southern Central Asia, the leaves (cilantro) are the most valuable part of the plant today, while the dried and ground mericarps are essential components for meat dishes, like *kebab*, *shashlyk*, *kuurdak*, and *meatballs.*

Cumin was recorded in Central Asia for the first time in this study at Bukhara in a mineralized state from badrabs and tashnaus dated to the tenth–eleventh centuries AD. While the original geographic distribution of cumin is not known, it likely grew wild across much of southwest and southern Central Asia. Zohary et al. (2012) claim that wild forms of cumin occur in the central regions of Central Asia, and Kislev with colleagues (2004) argue that it grew over a wider area. The earliest archaeological remains of cumin were recovered in the eastern Mediterranean (Frumin et al. 2015). Cumin is present in assemblages of the second millennium BC in Syria (van Zeist and Vynckier 1984) and the first millennium BC in Jordan (Zohary et al. 2012). It became more widespread staring from the end of first millennium BC and beginning of the first millennium AD (van der Veen 2011; Zohary et al. 2012; Frumin et al. 2015).

Sumac has never before been archaeologically identified in Central Asia and was likely introduced from western Asia in the second half of the first millennium AD. Sumac cultivation could be tied to economic factors, such as for the Bukharan textile industry in the first millennium AD as a dying agent. Sumac seeds have been recovered from the Neolithic site of Çatalhöyük (7480–7080 cal. BC) (Fairbairn et al. 2002) and at Bronze Age sites (ca. 3000–1200 BC) in Turkey, often accompanied by other fruits and spices (Fairbairn et al. 2019). Sumac remains were found on the Uluburum ship, dated to the fourteen and thirteen centuries BC in Greece (Bass 1997). However, it is far more likely that the Sumac was part of a spice.

## Conclusion

It appears that urban expansion and commerce facilitated the introduction of new varieties of fruits, annual crops, and spices. Fruits might have been grown in the Bukhara Oasis or in neighboring regions and may have been transported dried or fermented. However, the majority of specimens likely represent local cultivation since the agricultural economy of the Bukhara Oasis was historically based on cereal, cotton, and fruit. The Bukhara assemblage provides four perennial crops that have never before been identified in the Bukhara Oasis: figs, mulberries, sumac, and pomegranates (the last one being identified in art historical sources prior). Most of the fruit remains recovered in the Bukhara assemblage were likely grown in foothill orchards and possibly local gardens. Due to the preservation bias, we avoid comparing materials coming from the cultural deposits dated to the period before the Islamic Conquest (fourth–beginning of the eighth centuries AD) and materials dated to the Islamic period (tenth–twelfth centuries AD). While further research is needed, we speculate that the great quantity of fig, mulberry, pomegranate, melon, and sumac remains suggests that they were cultivated locally in the tenth–eleventh centuries AD.

## Supplementary Information

Below is the link to the electronic supplementary material.

Supplementary file1 (DOCX 19.8 kb)

Supplementary file2 (XLSX 52.9 kb)

Supplementary file3 (XLSX 31.8 kb)

Supplementary file4 (DOCX 15 kb)

## Data Availability

All data generated and analyzed during this study are included in this published article (and its supplementary information files). Sorted, photographed, and measured seeds are stored at the Max Planck Institute of Geoanthropology (in the future, contact the director of the Paleoethnobotany Laboratories at MPI-GA — Robert N. Spengler III).
