# Supplementary material for: Food globalization in southern Central Asia: archaeobotany at Bukhara between antiquity and the Middle Ages
Source: Archaeol Anthropol Sci. 2023 Jul 21;15(8):124. doi: 10.1007/s12520-023-01827-z (PMC10361866; doi:10.1007/s12520-023-01827-z)
Supplement: Supplementary file 1 — Supplementary file1 (DOCX 19.8 kb) [file 12520_2023_1827_MOESM1_ESM.docx]

Online Source 1

**Food Globalization in southern Central Asia: Archaeobotany at Bukhara between Antiquity and the Middle Ages**

Mir-Makhamad, Basira^1,2,3*^; Sören Stark^4^; Sirojidin Mirzaakhmedov^5^; Husniddin Rahmonov^5^; and Robert N. Spengler III^1,2^

1. Department of Archaeology, Max Planck Institute of Geoanthropology, Jena, Germany
2. Domestication and Anthropogenic Evolution Research Group, Max Planck Institute of Geoanthropology, Jena, Germany
3. Ancient Oriental Studies Department, Friedrich Schiller University, Jena, Germany
4. Institute for the Study of the Ancient World at New York University, New York, N.Y., USA
5. Samarkand Institute of Archaeology, Agency of Cultural Heritage of the Republic of Uzbekistan, Samarkand, Uzbekistan

| **Sample #** | **Context** | | **Sub-context** | **Conventional dates** | **14C (95ю4%)** |
| --- | --- | --- | --- | --- | --- |
|  | | **2020** | | | |
| FSB1 | Trench 1, ON18 (depth-60 cm) | | Building remains | 10^th^ century AD |  |
| FSB2 | Trench 2 (depth -42cm) | | Building remains | 10^th^ century AD |  |
| FSB3 | Trench 1, ON4 | | Tanur | 11^th^ – 12^th^ century AD | AD 261 – 532 |
|  |  |  |  |  | AD 255 – 416 |
| FSB4 | Trench 1 (depth -70cm) | | Building remains | 10^th^ century AD |  |
| FSB5 | Trench 3 (ON14) | | Hearth | 10^th^ – 11^th^ century AD |  |
| FSB6 | Trench 4 | | Layer with charcoal | NA |  |
| FSB7 | Trench 4 - (ON42) | | Badrab | 10^th^ – 11^th^ century AD |  |
| FSB8 | Trench 4 (ON28, depth - 40cm) | | Cultural layer | 10^th^ – 11^th^ century AD |  |
| FSB9 | Trench 4 - (ON39 - middle layer) | | Badrab | 10^th^ – 11^th^ century AD |  |
| FSB10 | Trench 4- (ON20, bellow FSB9) | | Badrab | 10^th^ – 11^th^ century AD | AD 882 – 991 |
| FSB11 | Trench 4- (ON35) | | Badrab | 10^th^ – 11^th^ century AD |  |
| FSB12 | Trench 1 (ON5, insitu - khum) | | Tanur | 11^th^ – 12^th^ century AD |  |
| FSB13 | Trench 1 (ON3) | | Tashnau | 10^th^ – 11^th^ century AD |  |
| FSB14 | Trench 1 (ON22) | | Tashnau | 10^th^ – 11^th^ century AD |  |
| FSB15 | Trench 1 (ON24, depth -2.5 m) | | Hearth | 6^th^ – 7^th^ century AD |  |
| FSB16 | Trench 4 (ON33) | | Tanur | 10^th^ century AD | AD 663 – 775 |
| FSB17 | Trench 1 (ON5, insitu - khum) | | Tanur | 11^th^ – 12^th^ century AD |  |
| FSB18 | Trench 4 -cesspit (ON44, depth - 7m) | | Badrab | 10^th^ – 11^th^ century AD |  |
| FSB19 | Trench 3 (ON14) | | Hearth | 10^th^ – 11^th^ century AD |  |
| FSB20 | R2 (depth - 1m) | | Layer with charcoal | NA |  |
| FSB21 | Trench 2019 - (ON29, depth - 3m) | | Badrab | 10^th^ – 11^th^ century AD |  |
| FSB22 | Trench 1 (depth - 6.2-6.8 m) | | Alluvial deposits | 3^rd^ cent. BC – 1^st^ cent. AD |  |
| FSB23 | Trench 4 - (ON44, depth - 7.2) | | Badrab | 10^th^ – 11^th^ century AD |  |
| FSB25 | Trench 4 - (ON44, depth - 7m) | | Badrab | 10^th^ – 11^th^ century AD |  |
| FSB26 | Trench 4 - (ON44, depth - 6.4 m) | | Badrab | 10^th^ – 11^th^ century AD |  |
| FSB27 | Trench 4 - (ON44, depth -7.2m) | | Badrab | 10^th^ – 11^th^ century AD |  |
|  | | **2021** | | | |
| FS1-21 | Trench 2 -cesspit (ON107, depth - 4-5m) | | Badrab | 10^th^ – 11^th^ century AD |  |
| FS2-21 | Trench 4 - cesspit 9ON 86, depth - 3m) | | Badrab | 10^th^ – 11^th^ century AD |  |
| FS3-21 | Trench 2 - (depth - 3 m) | |  | NA |  |
| FS4-21 | Trench 4 (depth - 1.5m) | | Cultural layers | 6^th^ – 8^th^ century AD |  |
| FS5-21 | Trench 4 - cesspit (ON 88, depth - 1m) | | Badrab | 10^th^ – 11^th^ century AD |  |
| FS6-21 | Trench 2 - cesspit (ON18, depth - 3m) | | Badrab | 10^th^ – 11^th^ century AD |  |
| FS7-21 | Trench 4 - cesspit (ON91) | | Badrab | 10^th^ – 11^th^ century AD |  |
| FS8-21 | Trench 4 - cesspit (ON82) | | Badrab | 10^th^ – 11^th^ century AD |  |
| FS9-21 | Trench 4 (depth - 1m) | | Cultural layers | 6^th^ – 8^th^ century AD |  |
| FS10-21 | Trench 2 - cesspit (depth - 1m) | | Badrab | 10^th^ – 11^th^ century AD |  |
| FS11-21 | Trench 5 (ON72) | | Hearth | 14^th^ – 15^th^ century AD |  |
| FS12-21 | Trench 5 (ON73) | | Hearth | 14^th^ – 15^th^ century AD |  |
| FS13-21 | Trench 5 (ON80, room 3) | | Filling of Room | 14^th^ – 15^th^ century AD |  |
| FS14-21 | Trench 4 | | Insitu from a pot | NA |  |
