# Supplementary material for: Food globalization in southern Central Asia: archaeobotany at Bukhara between antiquity and the Middle Ages
Source: Archaeol Anthropol Sci. 2023 Jul 21;15(8):124. doi: 10.1007/s12520-023-01827-z (PMC10361866; doi:10.1007/s12520-023-01827-z)
Supplement: Supplementary file 4 — Supplementary file4 (DOCX 15 kb) [file 12520_2023_1827_MOESM4_ESM.docx]

Online Source 4

**Food Globalization in southern Central Asia: Archaeobotany at Bukhara between Antiquity and the Middle Ages**

Mir-Makhamad, Basira^1,2,3*^; Sören Stark^4^; Sirojidin Mirzaakhmedov^5^; Husniddin Rahmonov^5^; and Robert N. Spengler III^1,2^

1. Department of Archaeology, Max Planck Institute of Geoanthropology, Jena, Germany
2. Domestication and Anthropogenic Evolution Research Group, Max Planck Institute of Geoanthropology, Jena, Germany
3. Ancient Oriental Studies Department, Friedrich Schiller University, Jena, Germany
4. Institute for the Study of the Ancient World at New York University, New York, N.Y., USA
5. Samarkand Institute of Archaeology, Agency of Cultural Heritage of the Republic of Uzbekistan, Samarkand, Uzbekistan

List of archaeological sites in Central Asia mentioned in the text and their GPS coordinates

| Site | GPS coordinates | |
| --- | --- | --- |
| Afrasiab (old Samarkand) | 39° 40' 12.792" | 66° 59' 15.8892" |
| Ak-Tobe 2 | 43° 13' 40.1196" | 74° 3' 1.1376" |
| Balalyk-Tepe | 37° 32' 7.3824" | 67° 6' 55.458" |
| Bazar-Dara | 38° 1' 31.0008" | 73° 18' 37.0008" |
| Bukhara | 39° 46' 36.228" | 64° 24' 46.8396 |
| Erk-Kala | 37° 40' 11.532" | 62° 11' 33.18" |
| Karakorum | 47° 12' 5.6664" | 102° 50' 34.1844" |
| Kafir-Kala | 39° 34' 19.1856" | 67° 1' 18.0228" |
| Kara-Tepe | 41° 53' 9.4524" | 60° 39' 56.556" |
| Krasnay-Rechka | 42° 54' 55.2024" | 75° 0' 35.7804" |
| Merv | 37° 40' 2.3376" | 62° 9' 24.4728" |
| Munguruk | 41° 17' 53.5164" | 69° 17' 9.0096" |
| Mugh | 39° 27' 3.4308" | 68° 24' 40.25484" |
| Novopokrovka 2 | 42° 52' 17.4432" | 74° 43' 22.764" |
| Panjakent | 39° 29' 13.9164" | 67° 37' 15.0384" |
| Paykend | 39° 35' 5.3232" | 64° 0' 22.3956" |
| Tashbulak | 39° 43' 19.7184" | 67° 49' 1.2684" |
| Taraz | 42° 54' 23.2128" | 71° 16' 47.9424" |
| Termez | 37° 15' 52.1712" | 67° 11' 28.7772" |
